# Sequence similarity of SARS-CoV-2 and humans: Implications for SARS-CoV-2 detection

**DOI:** 10.3389/fgene.2022.946359

**Published:** 2022-07-22

**Authors:** Heng Li, Xiaoping Hong, Liping Ding, Shuhui Meng, Rui Liao, Zhenyou Jiang, Dongzhou Liu

**Affiliations:** ^1^ Department of Rheumatology and Immunology, Shenzhen People’s Hospital, The Second Clinical Medical College of Jinan University, Shenzhen, China; ^2^ Integrated Chinese and Western Medicine Postdoctoral Research Station, Jinan University, Guangzhou, China; ^3^ Department of Microbiology and Immunology, School of Medicine, Jinan University, Guangzhou, China

**Keywords:** coronavirus, SARS-CoV-2 detection, mutation, COVID-19, coronavirus-COVID-19

## Abstract

Detecting severe acute respiratory syndrome coronavirus 2 (SARS-CoV-2) needs human samples, which inevitably contain trace human DNA and RNA. Sequence similarity may cause invalid detection results; however, there is still a lack of gene similarity analysis of SARS-CoV-2 and humans. All publicly reported complete genome assemblies in the Entrez genome database were collected for multiple sequence alignment, similarity and phylogenetic analysis. The complete genomes showed high similarity (>99.88% sequence identity). Phylogenetic analysis divided these viruses into three major clades with significant geographic group effects. Viruses from the United States showed considerable variability. Sequence similarity analysis revealed that SARS-CoV-2 has 612 similar sequences with the human genome and 100 similar sequences with the human transcriptome. The sequence characteristics and genome distribution of these similar sequences were confirmed. The sequence similarity and evolutionary mutations provide indispensable references for dynamic updates of SARS-CoV-2 detection primers and methods.

## Introduction

SARS-CoV-2 was first reported in December 2019 (McDonald 2021) and has spread rapidly worldwide, bringing severe social and economic problems to many countries (Biskanaki, Rallis et al., 2020; Miller, Becker et al., 2020; Castro, Kim et al., 2021). By June 2021, the SARS-CoV-2 pandemic had swollen to more than 170 million confirmed cases with a mortality rate of 2–3.4% (Yan et al., 2020a; Szcześniak, Gładka et al., 2021). High genetic infectivity, a large percent of asymptomatic cases and variability were major drivers of the epidemic (He, Qin et al., 2020; Wei, Geng et al., 2020). The reproduction number (*R*_0_) of SARS-CoV-2 was calculated to be 5.34 times higher than in SARS-CoV (3.1/0.58), and its latency was longer (Abdelrahman, Li et al., 2020). Generally, one infected patient can cause up to 5.7 further confirmed cases (Yan et al., 2020b; Kucharski, Russell et al., 2020; Wu, Leung et al., 2020). In addition, the infection of medical staff causes enormous losses of medical resources (Rongqing, Li et al., 2020; Liu, Ouyang et al., 2021), which worsens the pandemic. As a positive-sense (+) ssRNA-enveloped virus, the genome of SARS-CoV-2 is highly variable, and many mutant strains have been reported from different countries (including N501Y, D614G) (Leung, Shum et al., 2021; Mohammad, Alshawaf et al., 2021; Odeyale et al., 2021).

Viral mutations can affect the detection of SARS-CoV-2 (Peñarrubia, Ruiz et al., 2020; Dong, Wang et al., 2021; Hasan, Hossain et al., 2021). Currently, mainstream detection methods are based on the specificity of SARS-CoV-2 sequences. However, the specificity of commonly used detection primers for SARS-CoV-2 variants of concern remains unclear. The coronavirus genome is a single-stranded, positive-sense RNA ranging from 26 to 32 kilobases. SARS-CoV-2, severe acute respiratory syndrome (SARS) coronavirus and the Middle East respiratory syndrome (MERS) coronavirus are potentially lethal to humans among various coronaviruses (Drosten, Günther et al., 2003; Rota, Oberste et al., 2003; Zumla, Hui et al., 2015). From November 2002 to July 2003, SARS-CoV-1 coronavirus was responsible for more than 8000 cumulative infections (Prompetchara, Ketloy et al., 2020) and 774 deaths (9.6%) in 37 countries (Peiris, Yuen et al., 2003). MERS coronavirus has caused 2494 infections (Jiang, Xia et al., 2020) and 858 known deaths (35%) since September 2012 (https://www.who.int/emergencies/mers-cov/en/). The prevalence and lethality of coronaviruses pose a significant threat to human beings. Novel viral mutations could cause the failure of virus detection and the invalidity of vaccines. Gene sequence alignment of SARS-CoV-2 and humans is still absent in previous studies.

Mastering viral gene signatures and trends in genetic changes are necessary and ongoing efforts to maintain the dynamic update of viral detection methods and avoid viral detection escape. Although there were 998,314 nucleotide sequences related to SARS-CoV-2 in the NCBI Virus database by 01 November 2021 (https://www.ncbi.nlm.nih.gov/sars-cov-2/), only 92 sequences are recorded in the genomic form. We report the genetic relationship of 92 available genomes of SARS-CoV-2. The distribution of mutation sites was determined by multiple sequence alignments and constructed an evolutionary tree. The similarity of SARS-CoV-2 and human genes was quantified. This study provides essential information about the evolution and detection of SARS-CoV-2 from a new perspective.

## Materials and methods

### Data sources

This study analyzed all the SARS-CoV-2 genome assemblies publicly available in the Entrez genome databases by 01 November 2021 (https://www.ncbi.nlm.nih.gov/genome). By searching for " Severe acute respiratory syndrome coronavirus 2″, we collected 92 complete genome sequences. The related data were downloaded from GenBank (https://www.ncbi.nlm.nih.gov/genbank/). Information on viruses, such as genome size, GC, accession, CDS, release date, GenBank FTP resources, etc., are present in Supplementary Table S1.

### Genome analysis and comparison

ClustalW software (version 2.0.10) was used for sequence alignment, using the slow alignment setting. Similar sequences between SARS-CoV-2 and the human genome were searched using BLASTN, with the human genome assembly GRCh38.p13 as reference (Annotation Release 109.20200228) and MN908947.3 as a query. Word size (7), match/mismatch score (1, −1), and Gap costs (1,2) were used as parameters. The E value is 25, excluding repeated sequences.

### Phylogenetic analysis

Phylogenetic analysis of the complete SARS-CoV-2 genomes was conducted using MEGA software (version 7.0.14) with 1000 bootstrap replicates, employing the Fast Minimum Evolution method.

### Statistical analysis

Statistical analysis was conducted using SPSS 17.0 software (SPSS, Chicago, United States). The t-test was applied while comparing groups. The significance level was set at *p* < 0.05. GraphPad Prism 5 was used to generate graphics.

## Results

### Sources and distribution of complete SARS-CoV-2 genomes

Although many viral sequences have been reported, they are all presented as gene segments rather than genomic data. We collected 92 SARS-CoV-2 genome assemblies reported publicly in the Entrez genome database by 01 November 2021. All the genome sequences were complete, ranging from 29782 to 29903 nt in length. The related information is presented in Supplementary Table S1, including genome accession number, GC%, CDS, release date and GenBank resources.

**FIGURE 1:**
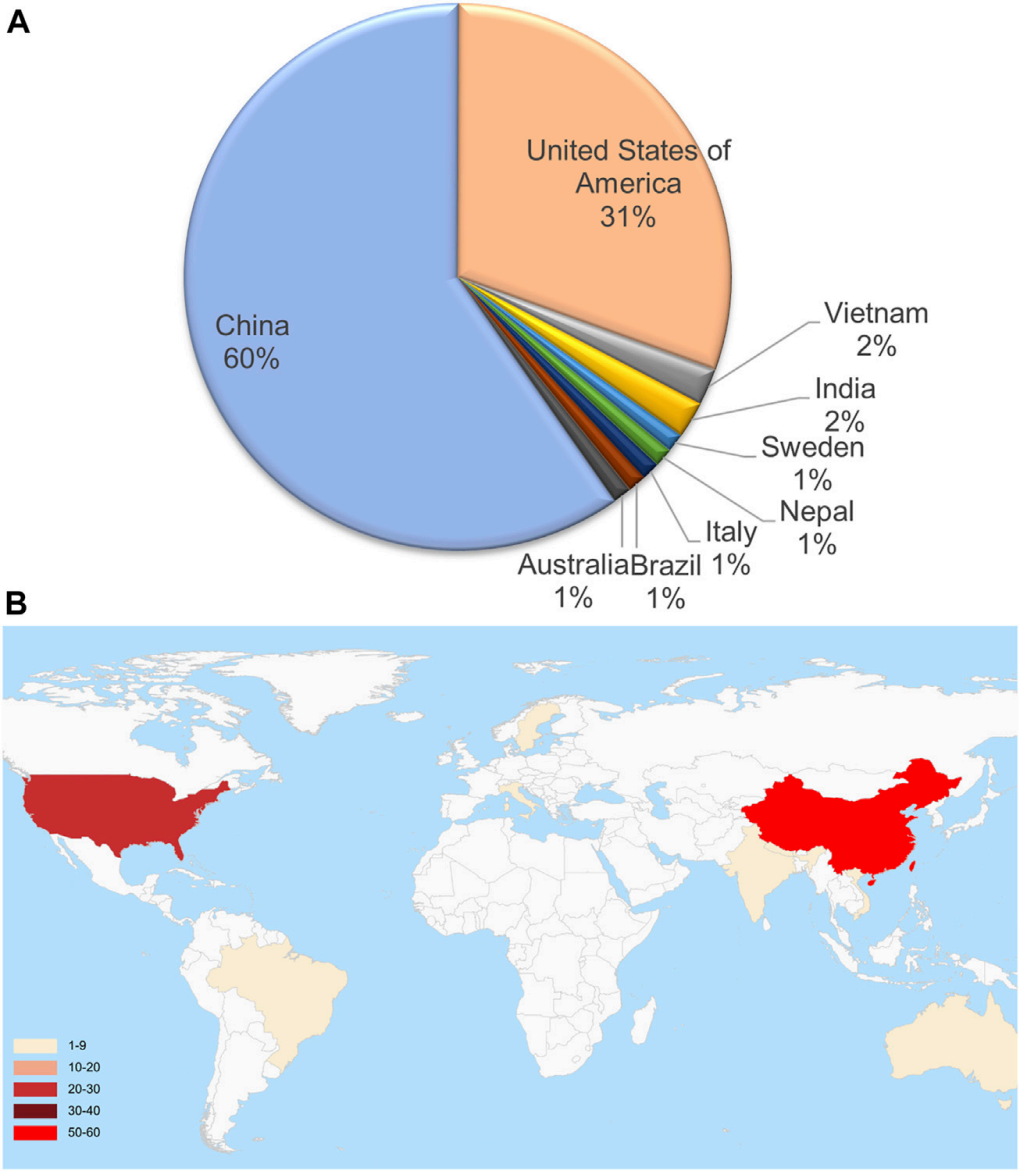
The proportion **(A)** and geographic locations **(B)** of 92 full-length sequenced SARS-CoV-2 genome assemblies.

**FIGURE 2:**
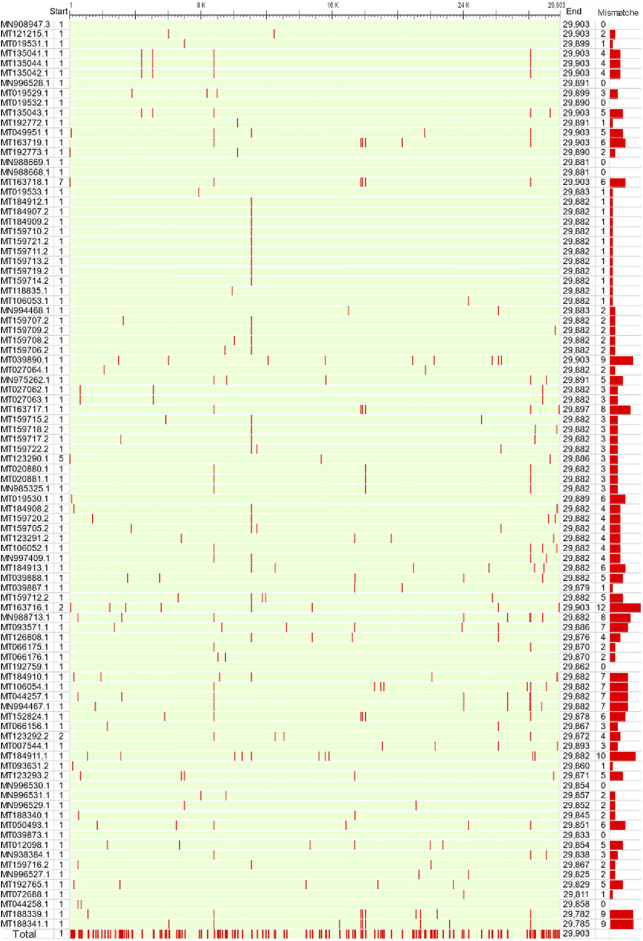
Sequence alignment of 92 full-length SARS-CoV-2 genomes. The first reported genome (MN908947.3) in Wuhan city was used as a reference. The bar on the right represents the total number of mutated bases for each genome. The bottom line converged all the mutation sites of 92 SARS-CoV-2 genomes.

These genome samples came from 9 countries. However, 60% were from China, 31% were from the United States, and only 9.8% were from other countries (Figure 1A). These countries are scattered around the world without apparent aggregation. Although a few countries are geographically contiguous, due to the barrier of mountains and rivers, the exchange of travelers is mainly dependent on airports (Figure 1B). The complete genome MN908947.3 from Wuhan city, first reported in March 2020, served as a reference. The sequences of these genomes are highly similar, and the sequence identity is higher than 99.88% (Figure 2A). The number of mutation sites was 0–12, with an average of 3.41.

### Sequence alignment revealed the mutation frequency of SARS-CoV-2 genomes

**FIGURE 3:**
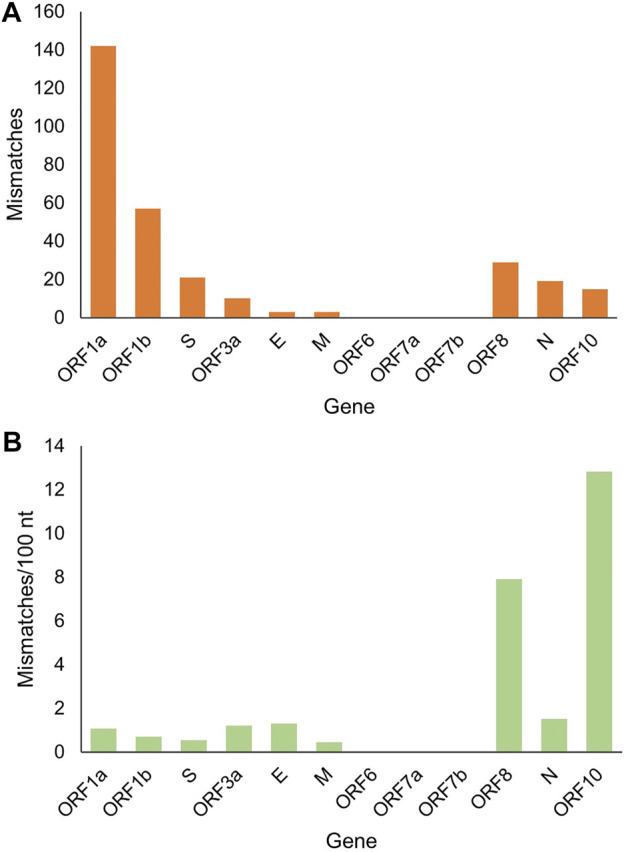
The landscape of gene mutations in the analyzed SARS-CoV-2 genomes. **(A)** The number of mismatched base pairs in each gene. **(B)** Mismatch rate in each gene.

It is worth noting that there were 299 mutation sites in these genomes in total, with an average of one mutation site per 100 nt. The largest sequence stretch without recorded mutations was ∼1000 nt. To clarify the landscape of individual genes, we performed a statistical analysis of the number and frequency of mutation sites for each gene (Figure 3). ORF1a and ORF1b contain 16 nonstructural proteins. As expected, ORF1a and ORF1b had the largest number of mutation sites as the longest sequences. However, their mutation frequency (mismatch/100 nt) was not high. By contrast, ORF10 and ORF8 had the first and second highest mutation frequencies, but the number of mutation sites was lower due to the short length. Analysis of the coding region also showed no mutations in ORF6, ORF7a and ORF7b (Figure 1B), indicating highly conserved. Secondly, structural protein membrane (M) and spike (S) also had lower mutation rates. S-protein contains receptor binding domain mediating viral invasion into host cells (Hoffmann, Kleine-Weber et al., 2020). Finally, mutations outside those genes are equally of concern, as there are unknown genes with unidentified functions, and all genes are subject to dynamic evolution. For example, ORF3d was identified and characterized by Nelson et al. as a novel overlapping gene in SARS-CoV-2 (Nelson, Ardern et al., 2020).

### Phylogenetic analysis revealed the evolutionary relationship of SARS-CoV-2 genomes

**FIGURE 4:**
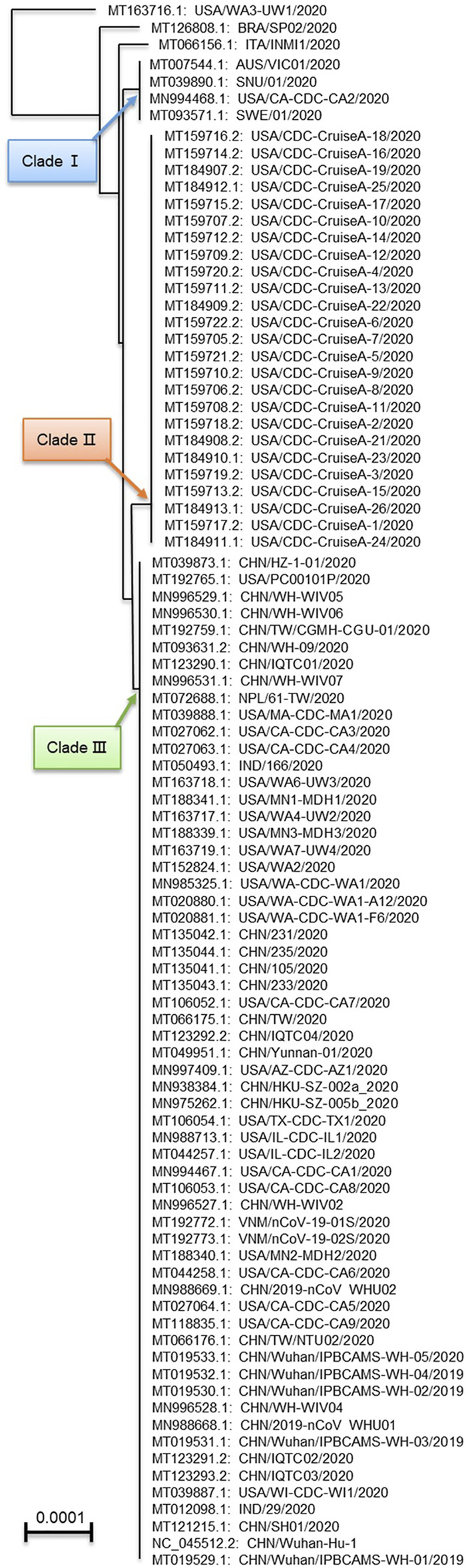
Phylogenetic analysis of full-length SARS-CoV-2 genomes.

Based on differences in genome sequences, we performed phylogenetic analysis of the SARS-CoV-2 genomes. The results showed three genomes (MT163716.1/USA/WA3-UW1/2020, MT126808.1 BRA/SP02/2020 and MT066156.1/ITA/INMI1/2020) formed independent branches (Figure 4). The remaining 89 genomes formed three major clades. Clade 1 contained only four genomes, and each of them was from a different country. Clade 2 contained 25 genomes, all from the United States CDC-Cruise A. Clade 3 contained the largest number of genomes, with 60 genomes from five countries. This evidence reveals the relationship and genetic distances between different mutant viruses.

### Characterization of sequence similarity of SARS-CoV-2 and human

**FIGURE 5:**
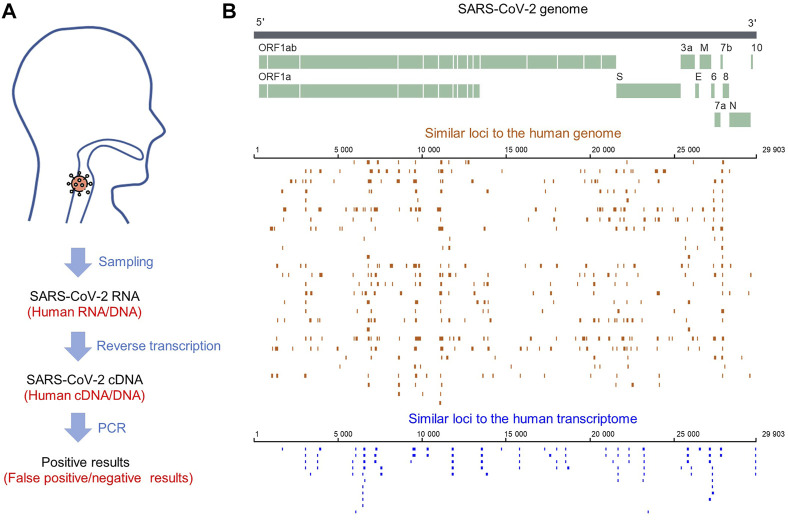
**(A)** SARS-CoV-2 sampling and processing flow. **(B)** Distribution of similar sequences between the SARS-CoV-2 genome and human genome/transcriptome.

**FIGURE 6:**
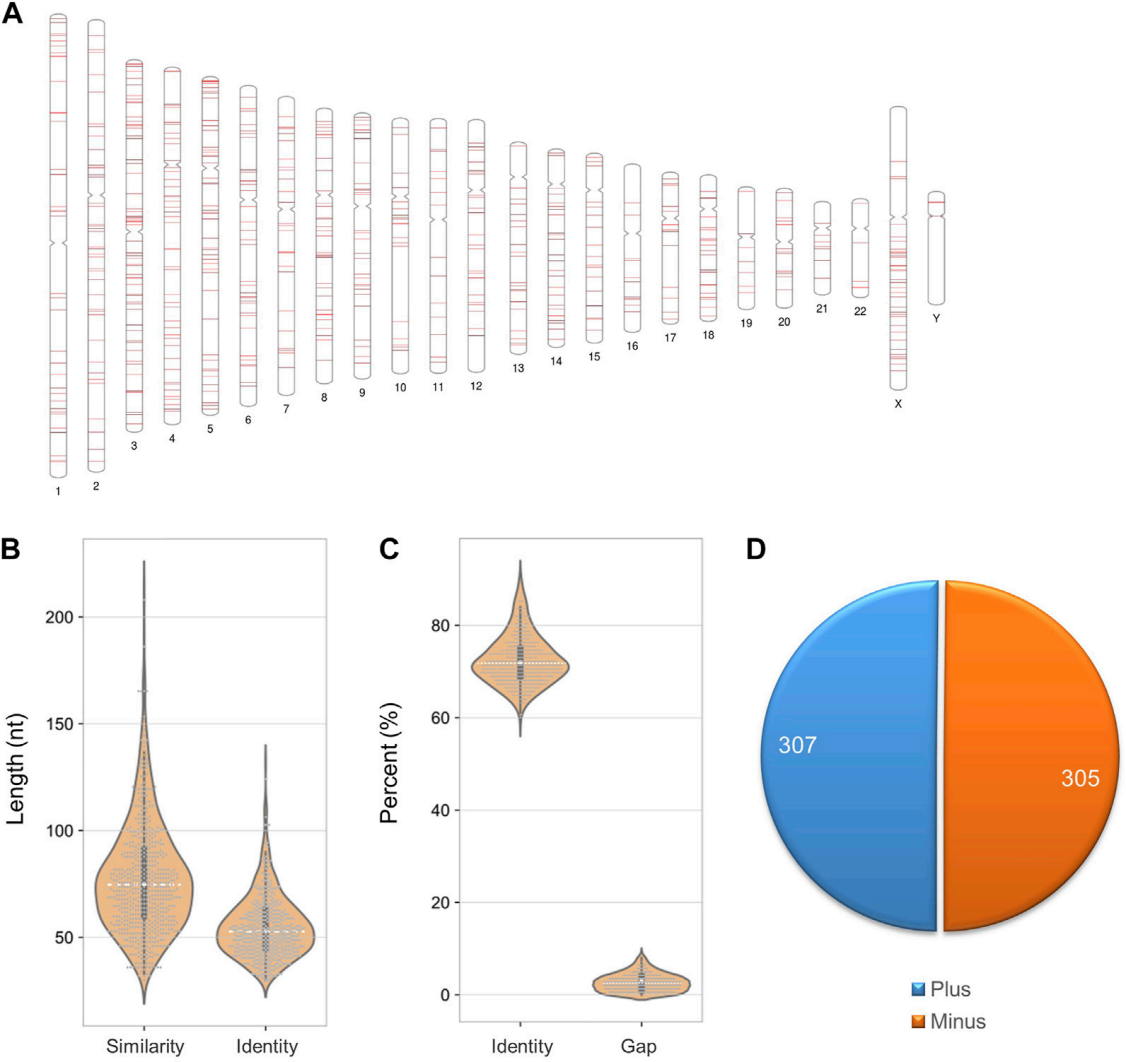
Sequence characteristics of similar loci between the SARS-CoV-2 and human genomes. **(A)** Distribution of similar sequences in the human chromosome. **(B)** Length of similar and consensus sequences. **(C)** The proportion of identity and gap. **(D)** Distribution of similar sequences in sense and antisense strands.

For SARS-CoV-2 detection, RNA needs to be reverse transcribed into DNA for sequencing, so foreign RNA/DNA could cause interference (Figure 5A). It has been reported that host (human) readings were mixed in the results of SARS-CoV-2 detection using patients’ bronchoalveolar lavage fluid samples or re-cultured viruses (Lu, Zhao et al., 2020). Since SARS-CoV-2 detection is mainly based on human samples, human genes are the primary interference source. After filtering the low complexity regions, we identified 612 similar sequences between SARS-CoV-2 and the human genome. The loci of these sequences are equally distributed in each chromosome, except for fewer on chromosomes Y, 22, 19, and 11 (Figure 6A). The similar sequences ranges in length from 33 to 212 nt, with an average sequence length of 77.9 nt and a median of 75 nt. The average sequence identity is 72.55%. The length of the consistent sequences ranges from 31 to 132 nt, with an average sequence length of 55.4 nt and a median of 53 nt (Figure 6B). The average gap rate was calculated to be 2.68%. These sequences are distributed in both the plus and minus strands at a ratio close to 1:1 (Figure 6C).

**FIGURE 7:**
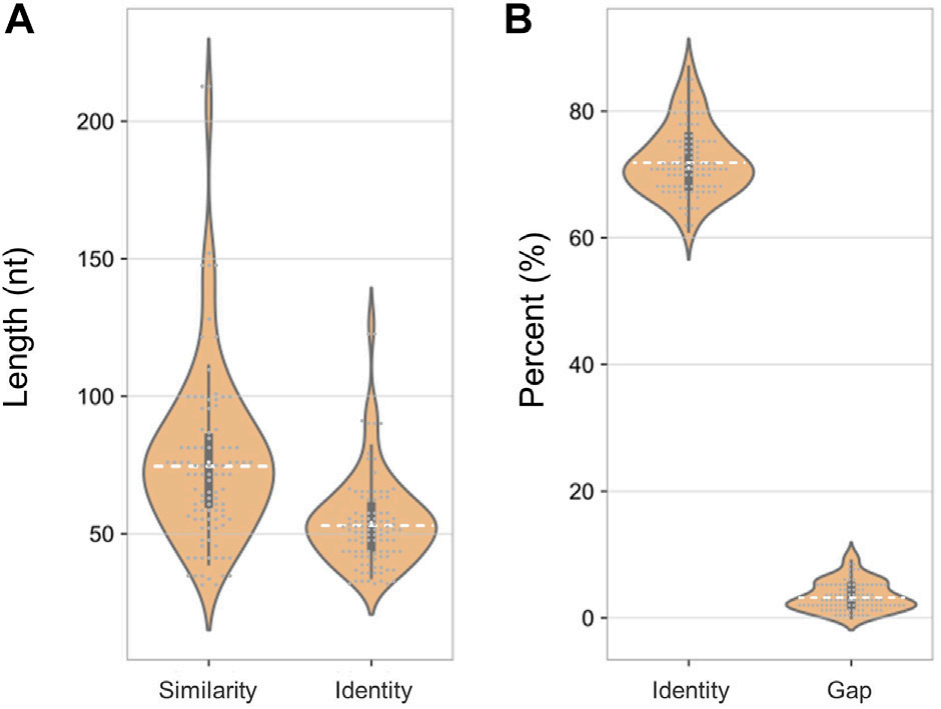
Sequence characteristics of similar loci between the SARS-CoV-2 genome and human transcriptome. **(A)** Length of similar and consensus sequences. **(B)** The proportion of identity and gap.

Notably, there were fewer similar sites in the human transcriptome than in the human genome (100 < 612), but their characteristics were consistent. The sequence similarity was close (72 vs. 71%) and the gap ratios were 3% for both (Figure 7). This result suggested that SARS-CoV-2 shares more sequence similarity with the human genome than with the transcriptome, indicating that admixed human DNA is more likely to affect the virus detection results and that there may be less interference from reverse transcription of human RNA.

We conducted enrichment analysis for genes where the similar sites were located. GO analysis enriched three significant terms: integral component of Golgi membrane (GO:0030173), vesicle-mediated transport to the plasma membrane (GO:0098876) and ubiquitin ligase complex (GO:0000151) respectively (*p* < 0.01). In contrast, KEGG analysis only obtained one pathway: estrogen signaling pathway (*p* < 0.01) (Supplementary Table S2). The results showed that these genes are not closely related.

## Discussion

Previous studies revealed that several viral mutations of SARS-CoV-2 may affect related detection and treatment strategies. Starr et al. showed that a single amino acid mutation in the receptor-binding domain (RBD) of SARS-CoV-2 entirely blocked the binding of the REGN-COV2 antibody (Starr, Greaney et al., 2021). Many mutants (E484K, N501Y and K417N) resulted in a more substantial loss of neutralizing activity of antibodies (Collier, De Marco et al., 2021; Tegally, Wilkinson et al., 2021; Wang, Schmidt et al., 2021). Specific mutants (E484K, T95I, del142-144, and D614G) were confirmed to cause vaccine breakthrough infections (Hacisuleyman, Hale et al., 2021). Multiple antibody combinations effectively protected against SARS-CoV-2 immune escape brought about by single-site mutations (Ku, Xie et al., 2021). However, not all mutations increase the risk of viral escape. For example, the D614G spike mutation increases SARS-CoV-2 susceptibility to neutralization by monoclonal antibodies and convalescent sera (Weissman, Alameh et al., 2021). The studies of the above mutants help us understand the challenges brought by virus mutation, but their objects are parts of the genome. Unlike previous studies on single amino acid or single-gene mutations, this study focuses on complete genome sequences, providing a landscape of SARS-CoV-2 genome mutations.

This study reports the alignment results and phylogenetic analysis of the existing complete SARS-CoV-2 genome sequences. Similarity analysis of SARS-CoV-2 and human whole genome/transcriptome sequences uncovered hundreds of similar sites. These results provide important information for SARS-CoV-2 detection and potential gene recombination possibilities.

Mutations are frequent in the genome of SARS-CoV-2, with an average of one mutation per 100 nt. However, base deletions are uncommon. Phylogenetic analysis indicated that except for the relatively unique genomes USA/WA3-UW1/2020, BRA/SP02/2020 and ITA/INMI1/2020 (MT163716.1, MT126808.1 and MT066156.1), the other SARS-CoV-2 genomes were divided into three clades. MT163716.1, MT126808.1 and MT066156.1 are from different countries, forming three separate branches at the base of the evolutionary tree. Clade Ⅰ includes four genomes from different countries and is at a certain evolutionary distance between them. Finally, clades Ⅱ and Ⅲ comprise most of the other genomes (including 25 and 60). From the base of the evolutionary tree (MT163716.1) to the three clades, all clades contain genomes from the United States. Based on current data, the genomes from the United States span the largest evolutionary distance. The virus similarity in each country is higher, indicating that the impact of travel restrictions is significant.

Subsequently, we analyzed similar sequences between the SARS-CoV-2 genome and the human genome/transcriptome. The analysis showed that SARS-CoV-2 has 612 similar sites to the human genome and 100 similar sites to the human transcriptome. We found that ∼70% of the similar sequences were completely identical and may influence detection primers. If the detection targets include these similar sites, the similar fragments may interfere with the Q-PCR results. The change of virus sequence may change the target site of detection, so it is necessary to carry out a genome-wide sequence comparison. Some commonly used SARS-CoV-2 detection primers are consistent with some human genes. For example, forward primer 1 ab: CCCTGTGGTTTACACTAA is consistent with the chromosome sequence 44831798 CCTGTGGGTTTACACT 44831814 of Homo sapiens isolate CHM13 chromosome 6 (sequence ID: NC_060930.1). The complementary fragments have the possibility of a mismatch in the PCR process. As viral genes mutate, the corresponding detection primers change. The detection targets need to avoid similar fragments to eliminate the interference caused by mismatches. Therefore, we believe that these sequences have the potential to interfere with viral detection and are not suitable as detection targets. Whether these similar viral sequences affect the expression or inheritance of human genes requires further investigation.

Our results suggest that the genomes of SARS-CoV-2 and humans contain many short similar sequences, with a sequence identity of ∼70%. The average length is 55.4 nt, long enough to contain detection primers. Thus, these sequences may cause interference in the process of virus detection. Although no recombination has been reported, sequence similarity provides a basis for recombination, and this inference may change if mixed sequences are identified.

SARS-CoV-2 has profoundly affected human society for several years. In turn, the rapid multiplication that comes with the pandemic accelerated its genetic variation. It is reported that SARS-CoV-2 could spread among animals, including pet cats and dogs (Chandler, Bevins et al., 2021; Dileepan, Di et al., 2021; Doerksen, Lu et al., 2021), and the wide range of hosts will increase its survivability. Gene interaction between virus and hosts is worth our vigilance, and the knowledge of sequence similarity is necessary to rule out spurious results to improve assay accuracy.

## Conclusion

This work investigates the geographical distribution, mutational characteristics and phylogenetic relationship of complete SARS-CoV-2 genomes. Several hundred similar gene sequences of SARS-CoV-2 and humans with high concordance were identified. The sequence length (median 75 nt) and sequence identity (72.55%) may potentially interfere with the binding of primers and templates in virus detection. Although SARS-CoV-2 genomic integration has not been reported, the risk of recombination through endogenous transposons warrants vigilance. The interference of these similar sequences with virus detection requires excellent attention, and the interaction and influence on human genes require further investigation.

## Data Availability

The original contributions presented in the study are included in the article/Supplementary Material, further inquiries can be directed to the corresponding authors.
